# 2-[(2*Z*,3*E*)-2-Hy­droxy­imino-5-phenyl-2,3-dihydro-3-thienyl­idene]-2-phenyl­acetonitrile

**DOI:** 10.1107/S1600536810024955

**Published:** 2010-07-07

**Authors:** Nazar Rad, Yuri Teslenko, Mykola Obushak, Volodymyr Pavlyuk, Bernard Marciniak

**Affiliations:** a Department of Chemistry, Ivan Franko National University of Lviv, Kyryla i, Mefodia Str. 8, 79005 Lviv, Ukraine; b Institute of Chemistry and Environment Protection Jan Dlugosz University of Czestochowa, al. Armii Krajowej 13/15, 42-200 Czestochowa, Poland

## Abstract

In the crystal structure of the title compound, C_18_H_12_N_2_OS, centrosymmetric dimers are stabilized both by van der Waals inter­actions and by two types of inter­molecular O—H⋯N hydrogen bonds. In addition, an intra­molecular C—H⋯S hydrogen bond is observed. The dihedral angles between the central ring and the two pendant phenyl rings are 7.4 (1) and 45.06 (9)°.

## Related literature

For related heterocyclic compounds, see: Suwinsky *et al.* (2003). For a similar benzooxime, see: Davis *et al.* (1960 ▶). For applications of related reaction conditions, see: Davis & Pizzini (1960 ▶); Davis *et al.* (1961 ▶). For supra­molecular chemistry based on oximes, see: Bertolasi *et al.* (1982 ▶); Chertanova *et al.* (1994 ▶). For the biological relevance of oximes and thio­phene derivatives, see: Rappoport & Liebman (2008 ▶); Gronowitz (1963 ▶). 
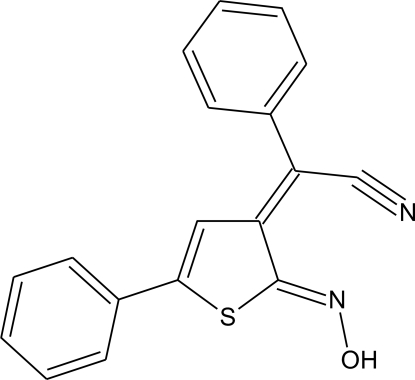


## Experimental

### 

#### Crystal data

C_18_H_12_N_2_OS
*M* *_r_* = 304.37
Monoclinic, 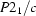

*a* = 7.9826 (5) Å
*b* = 21.3400 (7) Å
*c* = 8.7253 (5) Å
β = 90.471 (7)°
*V* = 1486.29 (14) Å^3^
*Z* = 4
Mo *K*α radiation
μ = 0.22 mm^−1^
*T* = 293 K
0.5 × 0.3 × 0.06 mm

#### Data collection

Oxford Diffraction Xcalibur3 CCD diffractometer
Absorption correction: multi-scan (*CrysAlis RED*; Oxford Diffraction, 2008 ▶) *T*_min_ = 0.909, *T*_max_ = 0.986
9461 measured reflections
3024 independent reflections
2240 reflections with *I* > 2σ(*I*)
*R*_int_ = 0.022

#### Refinement

*R*[*F* ^2^ > 2σ(*F* ^2^)] = 0.036
*wR*(*F* ^2^) = 0.104
*S* = 1.04
3024 reflections
203 parameters
H-atom parameters constrained
Δρ_max_ = 0.21 e Å^−3^
Δρ_min_ = −0.23 e Å^−3^

### 

Data collection: *CrysAlis CCD* (Oxford Diffraction, 2008 ▶); cell refinement: *CrysAlis CCD*; data reduction: *CrysAlis RED* (Oxford Diffraction, 2008 ▶); program(s) used to solve structure: *SHELXS97* (Sheldrick, 2008 ▶); program(s) used to refine structure: *SHELXL97* (Sheldrick, 2008 ▶); molecular graphics: *DIAMOND* (Brandenburg, 2006 ▶); software used to prepare material for publication: *SHELXL97* and *publCIF* (Westrip, 2010 ▶).

## Supplementary Material

Crystal structure: contains datablocks I, global. DOI: 10.1107/S1600536810024955/im2200sup1.cif

Structure factors: contains datablocks I. DOI: 10.1107/S1600536810024955/im2200Isup2.hkl

Additional supplementary materials:  crystallographic information; 3D view; checkCIF report

## Figures and Tables

**Table 1:** Hydrogen-bond geometry (Å, °)

*D*—H⋯*A*	*D*—H	H⋯*A*	*D*⋯*A*	*D*—H⋯*A*
O1—H1*A*⋯N1^i^	0.82	2.16	2.900 (2)	150
O1—H1*A*⋯N2^i^	0.82	2.40	2.888 (2)	119
C1—H1⋯S1	0.93	2.60	3.041 (2)	109

Symmetry code: (i) 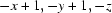.

**Fig. 1.**
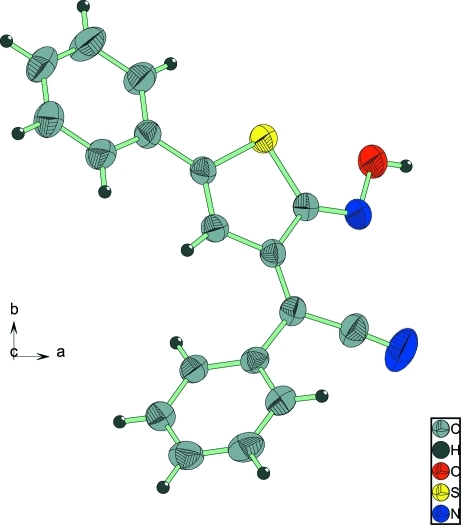
Molecular structure of the title compound, C18H12N2OS. Thermal ellipsoids represent a 50% probability level.

**Fig. 2.**
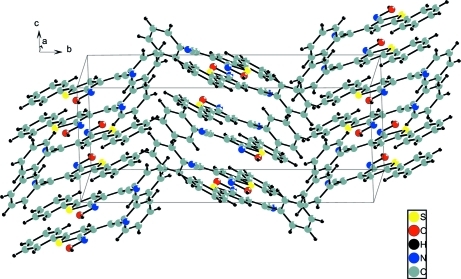
Crystal structure of the title compound.

## supplementary crystallographic information

### Comment

Oximes can act both as donors and acceptors for hydrogen bonds, making them interesting materials for supramolecular chemistry (Bertolasi *et al.*, 1982; Chertanova *et al.*, 1994). Besides, oximes are among the most useful and versatile intermediates in synthetic organic chemistry, the Beckmann rearrangement and the reduction of oximes being two of the most useful transformations. Oximes are also interesting due to their wide application in medicine, industry and analytical chemistry. Owing to the oxime bond oxime derivatives also posses therapeuthic efficacy as a chemical tool for targeted intracellular delivery of synthetic oligonucleotides *via* conjugation to cell-penetrating peptides (Rappoport & Liebman, 2008). On the other hand, the discoveries of thiophene compounds in fungi and higher plants has awakened the interest of the natural product chemist in the chemistry of thiophenes (Gronowitz, 1963) Synthesis of thiophen-oximes resulted from our interest in the investigation of reactions between nitro-thiophene derivatives and arylacetonitriles. Due to containing oximic and nitrile moieties the title compound may be useful in prospective modifications. The molecule of the title compound is not planar: the phenyl moiety neighbouring to nitrile group is deviated from planarity by 45.06 (9)° and the second phenyl moiety is twisted by 7.4 (1)°. In the crystal structure solely the *anti*-isomer of the oxime is observed. The components of the structure are united into a three dimensional network by an extensive system of O—H···N intermolecular hydrogen bonds next to the intramolecular C(1)—H(1)···S(1) hydrogen bond. Adjacent molecules are linked into dimers by intermolecular O—H···N hydrogen bonds under participation of oximic groups. The distance between the nitrile nitrogen and oximic hydrogen atom of another molecule is 2.403 (2) Å. Dimers are further stacked in columns along the unique axis by π-π stacking interactions with centroid-centroid distances of 3.6 (1) Å.

### Experimental

To 40 ml of a methanolic solution of potassium hydroxide (3.36 g, 60 mmoles) phenylacetonitrile (1.17 ml, 10 mmol) was added with stirring. Then 10 ml of a methanolic solution of 2-iodo-5-nitrothiophene (2.55 g, 10 mmol) was added to the reaction mixture. The suspension was stirred at room temperature until precipitation of product was ended. The reaction mixture was then poured into 100 ml of water and acidified by adding acetic acid. The precipitate was isolated by filtration, washed with water and dried. Red (orange) needles of title compound, m.p. (with decomp.) 394–395 K, yield 2.12 g (60%), were obtained after slowly cooling down an ethanolic solution.

### Refinement

Positions of H atoms were calculated and refined using SHELXL constraints. All H atoms, including one bonded to O, were positioned geometrically with O—H = 0.82 Å and with C—H = 0.93 Å. Finally, thermal parameters of all hydrogen atoms were refined using an overall thermal isotropic parameter excluding the hydrogen atom of OH-group. Thermal parameter for hydrogen of OH-group was refined individually.

### Crystal data

**Table:**

C_18_H_12_N_2_OS	*F*(000) = 632
*M**_r_* = 304.37	*D*_x_ = 1.360 Mg m^−^^3^
Monoclinic, *P*2_1_/*c*	Mo *K*α radiation, λ = 0.71073 Å
Hall symbol: -P 2ybc	Cell parameters from 2240 reflections
*a* = 7.9826 (5) Å	θ = 2.5–26.4°
*b* = 21.3400 (7) Å	µ = 0.22 mm^−^^1^
*c* = 8.7253 (5) Å	*T* = 293 K
β = 90.471 (7)°	Prism, translucent red
*V* = 1486.29 (14) Å^3^	0.5 × 0.3 × 0.06 mm
*Z* = 4	

### Data collection

**Table:**

Oxford Diffraction Xcalibur3 CCD diffractometer	3024 independent reflections
Radiation source: fine-focus sealed tube	2240 reflections with *I* > 2σ(*I*)
graphite	*R*_int_ = 0.022
ω scans	θ_max_ = 26.4°, θ_min_ = 2.5°
Absorption correction: multi-scan (*CrysAlis RED*; Oxford Diffraction, 2008)	*h* = −9→9
*T*_min_ = 0.909, *T*_max_ = 0.986	*k* = −26→26
9461 measured reflections	*l* = −6→10

### Refinement

**Table:**

Refinement on *F*^2^	Primary atom site location: structure-invariant direct methods
Least-squares matrix: full	Secondary atom site location: difference Fourier map
*R*[*F*^2^ > 2σ(*F*^2^)] = 0.036	Hydrogen site location: inferred from neighbouring sites
*wR*(*F*^2^) = 0.104	H-atom parameters constrained
*S* = 1.04	*w* = 1/[σ^2^(*F*_o_^2^) + (0.0639*P*)^2^] where *P* = (*F*_o_^2^ + 2*F*_c_^2^)/3
3024 reflections	(Δ/σ)_max_ = 0.004
203 parameters	Δρ_max_ = 0.21 e Å^−^^3^
0 restraints	Δρ_min_ = −0.23 e Å^−^^3^

### Special details

**Table:**

Geometry. All e.s.d.'s (except the e.s.d. in the dihedral angle between two l.s. planes) are estimated using the full covariance matrix. The cell e.s.d.'s are taken into account individually in the estimation of e.s.d.'s in distances, angles and torsion angles; correlations between e.s.d.'s in cell parameters are only used when they are defined by crystal symmetry. An approximate (isotropic) treatment of cell e.s.d.'s is used for estimating e.s.d.'s involving l.s. planes.
Refinement. Refinement of *F*^2^ against ALL reflections. The weighted *R*-factor *wR* and goodness of fit *S* are based on *F*^2^, conventional *R*-factors *R* are based on *F*, with *F* set to zero for negative *F*^2^. The threshold expression of *F*^2^ > σ(*F*^2^) is used only for calculating *R*-factors(gt) *etc*. and is not relevant to the choice of reflections for refinement. *R*-factors based on *F*^2^ are statistically about twice as large as those based on *F*, and *R*– factors based on ALL data will be even larger.

### Fractional atomic coordinates and isotropic or equivalent isotropic displacement parameters (Å^2^)

**Table:**

	*x*	*y*	*z*	*U*_iso_*/*U*_eq_	
S1	0.06721 (5)	0.580998 (19)	0.13057 (5)	0.04795 (16)	
O1	0.36433 (15)	0.55770 (6)	−0.00007 (15)	0.0580 (3)	
H1A	0.4546	0.5529	−0.0427	0.097 (8)*	
N1	0.32525 (16)	0.50389 (6)	0.08232 (15)	0.0450 (3)	
N2	0.4444 (2)	0.36514 (9)	0.2067 (2)	0.0826 (6)	
C1	−0.2697 (2)	0.64260 (8)	0.1895 (2)	0.0559 (5)	
H1	−0.1866	0.6575	0.1250	0.0632 (16)*	
C2	−0.4127 (3)	0.67764 (10)	0.2115 (2)	0.0680 (5)	
H2	−0.4250	0.7159	0.1614	0.0632 (16)*	
C3	−0.5363 (2)	0.65684 (9)	0.3058 (2)	0.0623 (5)	
H3	−0.6328	0.6805	0.3198	0.0632 (16)*	
C4	−0.5168 (2)	0.60064 (9)	0.3797 (2)	0.0628 (5)	
H4	−0.6001	0.5864	0.4448	0.0632 (16)*	
C5	−0.3749 (2)	0.56499 (9)	0.3585 (2)	0.0544 (5)	
H5	−0.3638	0.5268	0.4090	0.0632 (16)*	
C6	−0.24840 (18)	0.58563 (7)	0.26236 (17)	0.0408 (4)	
C7	−0.09669 (18)	0.54839 (7)	0.23836 (16)	0.0390 (4)	
C8	−0.06423 (19)	0.48957 (7)	0.28617 (18)	0.0415 (4)	
H8	−0.1393	0.4670	0.3456	0.0632 (16)*	
C9	0.18360 (19)	0.51144 (7)	0.14809 (17)	0.0388 (3)	
C10	0.09381 (18)	0.46385 (7)	0.23958 (16)	0.0375 (3)	
C11	0.14890 (19)	0.40472 (7)	0.27078 (16)	0.0391 (4)	
C12	0.3148 (2)	0.38504 (8)	0.2323 (2)	0.0523 (4)	
C13	0.04594 (19)	0.35461 (7)	0.34177 (17)	0.0378 (3)	
C14	−0.1207 (2)	0.34566 (7)	0.29922 (18)	0.0427 (4)	
H14	−0.1707	0.3724	0.2282	0.0632 (16)*	
C15	−0.2121 (2)	0.29741 (7)	0.36173 (19)	0.0491 (4)	
H15	−0.3234	0.2919	0.3324	0.0632 (16)*	
C16	−0.1409 (2)	0.25741 (8)	0.4668 (2)	0.0550 (5)	
H16	−0.2034	0.2248	0.5082	0.0632 (16)*	
C17	0.0237 (2)	0.26584 (8)	0.5107 (2)	0.0545 (4)	
H17	0.0718	0.2393	0.5833	0.0632 (16)*	
C18	0.1177 (2)	0.31341 (8)	0.44739 (18)	0.0468 (4)	
H18	0.2297	0.3180	0.4754	0.0632 (16)*	

### Atomic displacement parameters (Å^2^)

**Table:**

	*U*^11^	*U*^22^	*U*^33^	*U*^12^	*U*^13^	*U*^23^
S1	0.0387 (2)	0.0410 (2)	0.0644 (3)	0.00067 (16)	0.01095 (19)	0.00928 (19)
O1	0.0426 (7)	0.0559 (7)	0.0759 (9)	−0.0023 (5)	0.0200 (6)	0.0146 (6)
N1	0.0363 (7)	0.0461 (8)	0.0526 (8)	−0.0029 (6)	0.0073 (6)	0.0049 (6)
N2	0.0541 (11)	0.0943 (13)	0.0999 (14)	0.0288 (9)	0.0251 (9)	0.0426 (11)
C1	0.0530 (11)	0.0508 (10)	0.0639 (11)	0.0104 (8)	0.0127 (8)	0.0087 (9)
C2	0.0679 (13)	0.0587 (12)	0.0774 (13)	0.0254 (10)	0.0106 (10)	0.0133 (10)
C3	0.0495 (11)	0.0632 (12)	0.0743 (12)	0.0198 (9)	0.0064 (9)	−0.0050 (10)
C4	0.0458 (10)	0.0657 (12)	0.0774 (13)	0.0065 (9)	0.0213 (9)	−0.0022 (10)
C5	0.0466 (10)	0.0464 (9)	0.0706 (12)	0.0045 (8)	0.0148 (8)	0.0036 (8)
C6	0.0361 (8)	0.0401 (9)	0.0462 (9)	0.0009 (6)	0.0034 (7)	−0.0061 (7)
C7	0.0352 (8)	0.0386 (8)	0.0435 (8)	−0.0015 (6)	0.0050 (6)	−0.0032 (7)
C8	0.0390 (9)	0.0380 (8)	0.0476 (9)	−0.0012 (6)	0.0094 (7)	0.0020 (7)
C9	0.0336 (8)	0.0391 (8)	0.0439 (8)	−0.0002 (6)	0.0028 (6)	−0.0006 (7)
C10	0.0334 (8)	0.0399 (8)	0.0391 (8)	−0.0011 (6)	0.0030 (6)	−0.0012 (6)
C11	0.0348 (8)	0.0432 (8)	0.0396 (8)	0.0035 (6)	0.0023 (6)	0.0016 (6)
C12	0.0427 (10)	0.0565 (10)	0.0579 (11)	0.0085 (8)	0.0079 (8)	0.0176 (8)
C13	0.0407 (9)	0.0344 (8)	0.0385 (8)	0.0042 (6)	0.0067 (6)	−0.0013 (6)
C14	0.0421 (9)	0.0385 (8)	0.0476 (9)	0.0051 (7)	0.0025 (7)	0.0003 (7)
C15	0.0438 (10)	0.0427 (9)	0.0611 (11)	−0.0012 (7)	0.0063 (8)	−0.0017 (8)
C16	0.0628 (12)	0.0405 (9)	0.0619 (11)	−0.0011 (8)	0.0157 (9)	0.0045 (8)
C17	0.0671 (12)	0.0442 (9)	0.0522 (10)	0.0097 (8)	0.0025 (8)	0.0114 (8)
C18	0.0475 (10)	0.0435 (9)	0.0492 (9)	0.0067 (7)	−0.0013 (7)	0.0019 (7)

### Geometric parameters (Å, °)

**Table:**

S1—C9	1.7571 (15)	C7—C8	1.347 (2)
S1—C7	1.7609 (15)	C8—C10	1.438 (2)
O1—N1	1.3916 (17)	C8—H8	0.9300
O1—H1A	0.8200	C9—C10	1.480 (2)
N1—C9	1.282 (2)	C10—C11	1.363 (2)
N2—C12	1.142 (2)	C11—C12	1.432 (2)
C1—C2	1.379 (2)	C11—C13	1.487 (2)
C1—C6	1.382 (2)	C13—C14	1.391 (2)
C1—H1	0.9300	C13—C18	1.394 (2)
C2—C3	1.365 (3)	C14—C15	1.377 (2)
C2—H2	0.9300	C14—H14	0.9300
C3—C4	1.370 (3)	C15—C16	1.373 (2)
C3—H3	0.9300	C15—H15	0.9300
C4—C5	1.378 (2)	C16—C17	1.378 (2)
C4—H4	0.9300	C16—H16	0.9300
C5—C6	1.390 (2)	C17—C18	1.380 (2)
C5—H5	0.9300	C17—H17	0.9300
C6—C7	1.465 (2)	C18—H18	0.9300
			
C9—S1—C7	90.83 (7)	N1—C9—S1	122.34 (12)
N1—O1—H1A	109.5	C10—C9—S1	111.62 (11)
C9—N1—O1	109.24 (12)	C11—C10—C8	125.41 (13)
C2—C1—C6	120.82 (17)	C11—C10—C9	125.87 (13)
C2—C1—H1	119.6	C8—C10—C9	108.69 (13)
C6—C1—H1	119.6	C10—C11—C12	121.48 (14)
C3—C2—C1	120.71 (18)	C10—C11—C13	124.78 (13)
C3—C2—H2	119.6	C12—C11—C13	113.72 (13)
C1—C2—H2	119.6	N2—C12—C11	174.86 (18)
C2—C3—C4	119.24 (17)	C14—C13—C18	118.49 (14)
C2—C3—H3	120.4	C14—C13—C11	121.21 (13)
C4—C3—H3	120.4	C18—C13—C11	120.23 (14)
C3—C4—C5	120.74 (18)	C15—C14—C13	120.35 (15)
C3—C4—H4	119.6	C15—C14—H14	119.8
C5—C4—H4	119.6	C13—C14—H14	119.8
C4—C5—C6	120.52 (17)	C16—C15—C14	120.80 (16)
C4—C5—H5	119.7	C16—C15—H15	119.6
C6—C5—H5	119.7	C14—C15—H15	119.6
C1—C6—C5	117.97 (15)	C15—C16—C17	119.55 (16)
C1—C6—C7	120.69 (14)	C15—C16—H16	120.2
C5—C6—C7	121.34 (14)	C17—C16—H16	120.2
C8—C7—C6	128.19 (14)	C16—C17—C18	120.36 (16)
C8—C7—S1	113.06 (11)	C16—C17—H17	119.8
C6—C7—S1	118.74 (11)	C18—C17—H17	119.8
C7—C8—C10	115.79 (13)	C17—C18—C13	120.44 (16)
C7—C8—H8	122.1	C17—C18—H18	119.8
C10—C8—H8	122.1	C13—C18—H18	119.8
N1—C9—C10	125.99 (14)		
			
C6—C1—C2—C3	0.1 (3)	N1—C9—C10—C11	1.3 (3)
C1—C2—C3—C4	0.3 (3)	S1—C9—C10—C11	178.39 (13)
C2—C3—C4—C5	−0.6 (3)	N1—C9—C10—C8	−176.87 (15)
C3—C4—C5—C6	0.4 (3)	S1—C9—C10—C8	0.27 (16)
C2—C1—C6—C5	−0.3 (3)	C8—C10—C11—C12	−174.00 (15)
C2—C1—C6—C7	179.74 (18)	C9—C10—C11—C12	8.2 (2)
C4—C5—C6—C1	0.0 (3)	C8—C10—C11—C13	7.8 (2)
C4—C5—C6—C7	−179.99 (16)	C9—C10—C11—C13	−169.97 (14)
C1—C6—C7—C8	−172.02 (16)	C10—C11—C12—N2	177 (2)
C5—C6—C7—C8	8.0 (3)	C13—C11—C12—N2	−5(2)
C1—C6—C7—S1	6.3 (2)	C10—C11—C13—C14	41.3 (2)
C5—C6—C7—S1	−173.67 (13)	C12—C11—C13—C14	−137.00 (15)
C9—S1—C7—C8	0.99 (13)	C10—C11—C13—C18	−142.00 (16)
C9—S1—C7—C6	−177.60 (12)	C12—C11—C13—C18	39.7 (2)
C6—C7—C8—C10	177.39 (14)	C18—C13—C14—C15	0.6 (2)
S1—C7—C8—C10	−1.04 (18)	C11—C13—C14—C15	177.37 (14)
O1—N1—C9—C10	176.52 (13)	C13—C14—C15—C16	0.0 (2)
O1—N1—C9—S1	−0.34 (19)	C14—C15—C16—C17	0.3 (2)
C7—S1—C9—N1	176.57 (14)	C15—C16—C17—C18	−1.2 (3)
C7—S1—C9—C10	−0.69 (12)	C16—C17—C18—C13	1.9 (2)
C7—C8—C10—C11	−177.64 (15)	C14—C13—C18—C17	−1.5 (2)
C7—C8—C10—C9	0.49 (19)	C11—C13—C18—C17	−178.36 (14)

### Hydrogen-bond geometry (Å, °)

**Table:**

*D*—H···*A*	*D*—H	H···*A*	*D*···*A*	*D*—H···*A*
O1—H1A···N1^i^	0.82	2.16	2.900 (2)	150
O1—H1A···N2^i^	0.82	2.40	2.888 (2)	119
C1—H1···S1	0.93	2.60	3.041 (2)	109

Symmetry codes: (i) −*x*+1, −*y*+1, −*z*.
